# Supplementary material for: Modeling glioblastoma heterogeneity as a dynamic network of cell states
Source: Mol Syst Biol. 2021 Sep 16;17(9):e10105. doi: 10.15252/msb.202010105 (PMC8444284; doi:10.15252/msb.202010105)
Supplement: Supplementary file 6 — Source Data for Figure 5 [file MSB-17-e10105-s004.zip › Figure5A_sourcedata/GSEA_3017/nosigdb_stateC.GseaPreranked.1622795002785/RICHARDS_ZHONG_NATURE_2018_ASTROCYTES_UPREG.html]

Details for gene set RICHARDS\_ZHONG\_NATURE\_2018\_ASTROCYTES\_UPREG[GSEA]

|  || Dataset | state13017 |
| Phenotype | NoPhenotypeAvailable |
| Upregulated in class | na\_neg |
| GeneSet | RICHARDS\_ZHONG\_NATURE\_2018\_ASTROCYTES\_UPREG |
| Enrichment Score (ES) | -0.62310535 |
| Normalized Enrichment Score (NES) | -3.9775674 |
| Nominal p-value | 0.0 |
| FDR q-value | 0.0 |
| FWER p-Value | 0.0 |
Table: GSEA Results Summary

  

Fig 1: Enrichment plot: RICHARDS\_ZHONG\_NATURE\_2018\_ASTROCYTES\_UPREG      
 Profile of the Running ES Score & Positions of GeneSet Members on the Rank Ordered List

  

| PROBE | GENE SYMBOL | GENE\_TITLE | RANK IN GENE LIST | RANK METRIC SCORE | RUNNING ES | CORE ENRICHMENT || 1 | ETV1 |  |  | 44 | 0.438 | -0.0708 | No |
| 2 | FERMT2 |  |  | 227 | -0.259 | -0.4109 | No |
| 3 | ZFYVE21 |  |  | 247 | -0.263 | -0.4392 | No |
| 4 | GNG7 |  |  | 253 | -0.264 | -0.4407 | No |
| 5 | ID4 |  |  | 261 | -0.267 | -0.4459 | No |
| 6 | GATM |  |  | 272 | -0.270 | -0.4568 | No |
| 7 | HSD17B12 |  |  | 277 | -0.271 | -0.4562 | No |
| 8 | DTNA |  |  | 281 | -0.271 | -0.4537 | No |
| 9 | TSC22D4 |  |  | 303 | -0.279 | -0.4853 | No |
| 10 | SLC1A3 |  |  | 307 | -0.281 | -0.4825 | No |
| 11 | LIFR |  |  | 311 | -0.282 | -0.4797 | No |
| 12 | DHCR7 |  |  | 322 | -0.285 | -0.4901 | No |
| 13 | SPRY1 |  |  | 369 | -0.307 | -0.5687 | No |
| 14 | PDPN |  |  | 372 | -0.307 | -0.5631 | No |
| 15 | CSPG5 |  |  | 381 | -0.310 | -0.5690 | No |
| 16 | S100A13 |  |  | 396 | -0.319 | -0.5860 | No |
| 17 | PLTP |  |  | 397 | -0.319 | -0.5763 | No |
| 18 | TIMP1 |  |  | 399 | -0.320 | -0.5684 | No |
| 19 | ZFP36L2 |  |  | 401 | -0.321 | -0.5606 | No |
| 20 | SLC4A4 |  |  | 415 | -0.332 | -0.5753 | No |
| 21 | FAT1 |  |  | 441 | -0.346 | -0.6125 | Yes |
| 22 | RAB31 |  |  | 445 | -0.349 | -0.6077 | Yes |
| 23 | BCAN |  |  | 449 | -0.351 | -0.6027 | Yes |
| 24 | LUZP2 |  |  | 450 | -0.352 | -0.5919 | Yes |
| 25 | IDI1 |  |  | 451 | -0.353 | -0.5812 | Yes |
| 26 | SERPINE2 |  |  | 452 | -0.355 | -0.5704 | Yes |
| 27 | DKK3 |  |  | 454 | -0.356 | -0.5614 | Yes |
| 28 | RHOC |  |  | 455 | -0.359 | -0.5505 | Yes |
| 29 | SLITRK2 |  |  | 458 | -0.361 | -0.5433 | Yes |
| 30 | EDNRB |  |  | 462 | -0.367 | -0.5378 | Yes |
| 31 | SERPINB6 |  |  | 463 | -0.367 | -0.5266 | Yes |
| 32 | ALCAM |  |  | 467 | -0.370 | -0.5211 | Yes |
| 33 | B2M |  |  | 468 | -0.371 | -0.5098 | Yes |
| 34 | HES1 |  |  | 470 | -0.373 | -0.5003 | Yes |
| 35 | LGALS3 |  |  | 471 | -0.374 | -0.4889 | Yes |
| 36 | CYP51A1 |  |  | 476 | -0.378 | -0.4850 | Yes |
| 37 | C1orf61 |  |  | 491 | -0.396 | -0.4997 | Yes |
| 38 | FABP7 |  |  | 495 | -0.411 | -0.4929 | Yes |
| 39 | PLIN3 |  |  | 496 | -0.411 | -0.4804 | Yes |
| 40 | AGT |  |  | 497 | -0.412 | -0.4678 | Yes |
| 41 | NT5E |  |  | 500 | -0.416 | -0.4589 | Yes |
| 42 | JUN |  |  | 503 | -0.425 | -0.4498 | Yes |
| 43 | SCD |  |  | 504 | -0.431 | -0.4366 | Yes |
| 44 | F3 |  |  | 505 | -0.432 | -0.4234 | Yes |
| 45 | SLC25A18 |  |  | 507 | -0.433 | -0.4122 | Yes |
| 46 | SCRG1 |  |  | 508 | -0.434 | -0.3989 | Yes |
| 47 | DBI |  |  | 514 | -0.440 | -0.3951 | Yes |
| 48 | CD9 |  |  | 515 | -0.443 | -0.3815 | Yes |
| 49 | PTN |  |  | 517 | -0.446 | -0.3699 | Yes |
| 50 | CST3 |  |  | 520 | -0.452 | -0.3599 | Yes |
| 51 | MSMO1 |  |  | 525 | -0.460 | -0.3535 | Yes |
| 52 | HMGCS1 |  |  | 529 | -0.466 | -0.3450 | Yes |
| 53 | IFITM3 |  |  | 533 | -0.477 | -0.3362 | Yes |
| 54 | TTYH1 |  |  | 540 | -0.496 | -0.3326 | Yes |
| 55 | SEMA6D |  |  | 541 | -0.500 | -0.3173 | Yes |
| 56 | LRRN3 |  |  | 543 | -0.504 | -0.3039 | Yes |
| 57 | HOPX |  |  | 546 | -0.513 | -0.2920 | Yes |
| 58 | RAMP1 |  |  | 548 | -0.517 | -0.2782 | Yes |
| 59 | GJA1 |  |  | 552 | -0.527 | -0.2678 | Yes |
| 60 | TIMP3 |  |  | 553 | -0.530 | -0.2517 | Yes |
| 61 | SPARCL1 |  |  | 557 | -0.544 | -0.2408 | Yes |
| 62 | S100B |  |  | 561 | -0.561 | -0.2295 | Yes |
| 63 | TNC |  |  | 564 | -0.572 | -0.2159 | Yes |
| 64 | MT2A |  |  | 566 | -0.575 | -0.2002 | Yes |
| 65 | S100A16 |  |  | 569 | -0.582 | -0.1863 | Yes |
| 66 | ITPR2 |  |  | 572 | -0.634 | -0.1708 | Yes |
| 67 | GFAP |  |  | 574 | -0.659 | -0.1526 | Yes |
| 68 | VIM |  |  | 578 | -0.674 | -0.1378 | Yes |
| 69 | CLU |  |  | 581 | -0.690 | -0.1206 | Yes |
| 70 | ATP1B2 |  |  | 585 | -0.714 | -0.1045 | Yes |
| 71 | CA12 |  |  | 586 | -0.717 | -0.0827 | Yes |
| 72 | PMP22 |  |  | 590 | -0.837 | -0.0629 | Yes |
| 73 | MT3 |  |  | 591 | -0.837 | -0.0374 | Yes |
| 74 | FABP5 |  |  | 596 | -1.477 | 0.0000 | Yes |
Table: GSEA details [plain text format]

  

Fig 2: RICHARDS\_ZHONG\_NATURE\_2018\_ASTROCYTES\_UPREG: Random ES distribution      
 Gene set null distribution of ES for **RICHARDS\_ZHONG\_NATURE\_2018\_ASTROCYTES\_UPREG**
